# High Ancient Genetic Diversity of Human Lice, *Pediculus humanus*, from Israel Reveals New Insights into the Origin of Clade B Lice

**DOI:** 10.1371/journal.pone.0164659

**Published:** 2016-10-14

**Authors:** Nadia Amanzougaghene, Kosta Y. Mumcuoglu, Florence Fenollar, Shir Alfi, Gonca Yesilyurt, Didier Raoult, Oleg Mediannikov

**Affiliations:** 1 Unité de Recherche sur les Maladies Infectieuses Tropicales Emergentes (URMITE) UM63, CNRS 7278, IRD 198, INSERM 1095, Aix-Marseille Université, Marseille cedex 05, France; 2 Parasitology Unit, Department of Microbiology and Molecular Genetics, The Kuvin Center for the Study of Infectious and Tropical Diseases, Hadassah Medical School, The Hebrew University, Jerusalem, Israel; 3 Campus International UCAD-IRD, Dakar, Senegal; Tianjin University, CHINA

## Abstract

The human head louse, *Pediculus humanus capitis*, is subdivided into several significantly divergent mitochondrial haplogroups, each with particular geographical distributions. Historically, they are among the oldest human parasites, representing an excellent marker for tracking older events in human evolutionary history. In this study, ancient DNA analysis using real-time polymerase chain reaction (qPCR), combined with conventional PCR, was applied to the remains of twenty-four ancient head lice and their eggs from the Roman period which were recovered from Israel. The lice and eggs were found in three combs, one of which was recovered from archaeological excavations in the Hatzeva area of the Judean desert, and two of which found in Moa, in the Arava region, close to the Dead Sea. Results show that the head lice remains dating approximately to 2,000 years old have a *cytb* haplogroup A, which is worldwide in distribution, and haplogroup B, which has thus far only been found in contemporary lice from America, Europe, Australia and, most recently, Africa. More specifically, this haplogroup B has a B36 haplotype, the most common among B haplogroups, and has been present in America for at least 4,000 years. The present findings confirm that clade B lice existed, at least in the Middle East, prior to contacts between Native Americans and Europeans. These results support a Middle Eastern origin for clade B followed by its introduction into the New World with the early peoples. Lastly, the presence of *Acinetobacter baumannii* DNA was demonstrated by qPCR and sequencing in four head lice remains belonging to clade A.

## Introduction

The human louse, *Pediculus humanus*, is an obligatory haematophagous parasite that thrived exclusively on human blood for at least 5–7 million years ago [1, 2]. This species includes two ecotypes: the head louse (*Pediculus humanus capitis* De Geer) that lives and multiplies on the scalp, and the body louse (*Pediculus humanus humanus* Linnaeus), that lives and oviposits on clothes [3, 4, 5].

Until recently, only the body louse was assumed to act as a vector for at least three serious human diseases, namely epidemic typhus, trench fever and louse-borne relapsing fever caused by *Rickettsia prowazekii*, *Bartonella quintana*, and *Borrelia recurrentis*, respectively [6]. Body lice have also been shown to be able to host and possibly transmit the agent of plague, *Yersinia pestis* [5, 7]. Though head lice have been found in nature to carry the DNA of *Bartonella quintana*, *Borrelia recurrentis*, *Acinetobacter baumannii* and *Yersinia pestis* [5, 8, 9, 10, 11, 12, 13], and experimental infections have shown that these lice can also act as a vector of louse-borne diseases [14, 15], their epidemiological significance is still debated.

Mitochondrial DNA (mtDNA) has been widely used to study the genetic diversity of human lice, revealing the presence of several deeply divergent mtDNA clades or haplogroups named A, B and C [1, 2, 16, 17, 18]. Haplogroup A is the most common. It can be found throughout the world and includes both head and body lice [1, 2, 17, 18]. Clade B comprises only head lice. It is confined to the New Word, Europe and Australia and was recently reported from North and South Africa [2, 18, 19, 20]. Clade C, which only includes head lice, has been found in Ethiopia, Nepal and Thailand [13, 16]. Most recently, two additional novel clades were described in 2015 by Drali et al. and Ashfaq et al. [5, 20] besides the three classical recognized clades. The first novel clade is the clade D described by Drali et al. [5] and is referred as clade E in Ashfaq et al. [20]. This clade (clade D *sensu* Drali et al.) is the sister group of clade A and consist on lice from Ethiopia and Democratic Republic of the Congo and comprising both head and body lice [5]. The second novel clade is described only by Ashfaq et al. [20]. This clade is the sister group of clade C and consist on lice from Senegal and Mali, referred here as clade “E”. This clade comprises only head lice.

*Pediculus humanus* is probably one of the oldest and most intimate human parasites [21, 22] and is known to have a long-term co-evolutionary association with humans over millions of years [1]. As such it represents an excellent marker for tracking older events in human evolutionary history [4, 18].

Louse infestations are mentioned in the Bible as the third plague [21]. Lice have also been found in a variety of archaeological contexts around the word [21, 22, 23, 24]. These reports all indicate the long-time presence of lice throughout the world before the globalization initiated during Columbus’s era, as the result of early human migrants out of Africa [4]. For example, in the Old World, the earliest record of head lice goes back to the Neolithic age, roughly 9,000 years ago, obtained from an individual who lived in the Nahal Hemar cave in Israel [21]. In the New World, the oldest such record of head lice comes from an archaic human skeleton in Brazil, dated at more than 10,000 years old [22].

However, few molecular data on ancient lice are available. Indeed, ancient DNA offers a direct means to assess the past genetic structure and diversity of human lice, which can provide relevant information relating to the antiquity of migration patterns of humans, their lice and, therefore, the flow of louse-borne pathogens [25].

Thus, based on molecular analyses of head lice from Peruvian mummies, D. Raoult *et al*. showed that the worldwide clade A had a pre-Columbian presence in the American continent and likely had links to the Old World [17]. In 2013, A. Boutellis *et al*. confirmed this result and demonstrated that Clade B was also present in America for more than 4,000 years, prior to contact with Europe, suggesting an American origin for this haplogroup, followed by its dispersal to the Old World from America beginning in the 16^th^ century [26]. Despite this finding, the precise source of this haplogroup prior to globalization remains unclear. However, other studies have suggested that could originate from Asia, which is reported to have populated the Americas [1, 18].

The analysis of ancient head louse eggs recovered from Israel dating from the Chalcolithic and early Islamic period, showed that these eggs may have belonged to people originating from West Africa, based on identification of the louse mitochondrial sub-clade C specific to that region [25].

In the present study, ancient DNA analysis of head lice remains dating from Roman period recovered from Israel was undertaken in order to: (1) identify their mitochondrial phylotypes, (2) reveal the phylogenetic relationship between ancient and contemporary human lice, and (3) look for louse-borne pathogens in these remains.

## Materials and Methods

### Ancient Head Lice Remains

**Fig 1:**
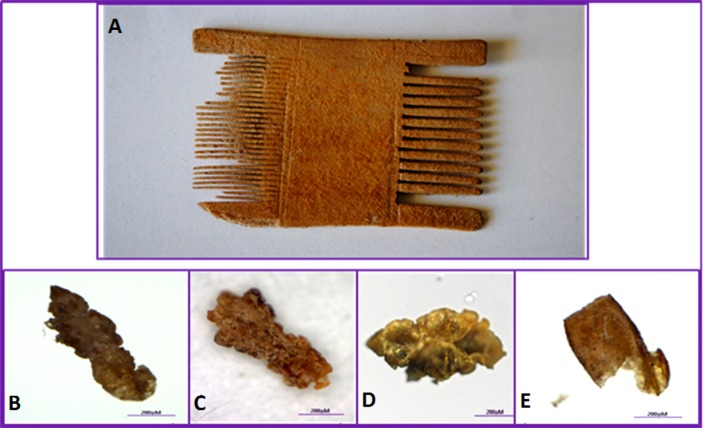
**Recovery of ancient human head lice from a two-sided louse comb belonging to the Roman period (A) recovered from the Judean desert and Arava regions of Israel**. In the lower part, entire specimens (B and C), the head and thorax of a head louse (D) and a damaged non-operculated egg (E) can be seen.

Head lice and their eggs were isolated from three louse combs from the Roman period (1^st^ century AD to 6^th^ century BC). Comb A and Comb B were recovered from archaeological excavations in Moa, in the Arava region, close to the Dead Sea, while Comb C was found in the Hatzeva area of the Judean desert (between Jericho and Dead Sea). In Moa, the excavations were conducted between 1981 and 1985 (permit no. A-1016/1981). The site appears to be a way station on the Spice Route connecting Petra to Gaza, where the remains of a caravanserai, a fortress and a temple were found. The excavation site in Hatzeva was a fortified road station from the Nabatean period in an agricultural settlement from the Early Arabic, late Roman period. The three combs were two-sided (Fig 1). From comb A, six lice parts and four eggs were isolated, from comb B, one entire specimen of male and nymph, respectively, eight lice parts and three eggs while from comb C only one entire nymph was isolated. All of the lice remains were preserved dry under sterile conditions. Morphological examination revealed that most of the eggs were embryonated. All examined combs are deposited in the Antiquities Authority in Jerusalem, Israel. Permission has been obtained from the Antiquities Authority to examine the combs and publish the results. All necessary permits were obtained for the described study, which complied with all relevant regulations.

### Ancient DNA Analysis

#### DNA extraction

Lice and eggs were rinsed twice in distilled water for 15 minutes and then crushed individually in sterile Eppendorf tubes. Two extractions blank were systematically co-extracted with the ancient lice samples during each extraction session. No more than five ancient samples were co-extracted at the same time. Finally, the total-DNA was extracted from seventeen head lice parts and seven nit/egg-parts using a phenol/chloroform/isoamyl protocol [27]. The extracted genomic DNA concentrations varied between 1.2 to 1.7 ng/ml for seven nit/egg-parts and between 1.4 to 2.4 ng/ml for seventeen head lice parts.

#### Identification of lice DNA and determination of lice clade

In order to decrease the possibility of contamination and false-positives, two sensitive sets of primers and probes for real-time quantitative polymerase chain reaction (qPCRs) were specifically designed for this study. Both qPCRs were designed in order to amplify all known clades of *P*. *humanus*, targeted 88-bp and 100-bp fragments of the cytochrome *b* gene (*cytb*) and the 12S ribosomal RNA (12S RNA), respectively. The design was performed with Primer3 software, version 4.0 (http://frodo.wi.mit.edu/primer3/), according to procedures described elsewhere [28]. The oligonucleotide sequences of the primers and probes were as follows: CytbF1 (5'- AGTGCTATTCCTRTTRTTGG-3'), CytbR1 (5'-AAYARYCGCTCTAAAGTAGG-3') and TaqMan probe (FAM-TGAGGAGGGTTTTCAGT- MGB) for the *cytb* gene, 12SF1 (5'- ATCTTACCTTTTAACTTTTGCT-3'), 12SR1 (5'- GCGTCTTGACTTGTACRTTA-3') and TaqMan probe (FAM- CTGGCACGTCGCTGTACTAA—MGB) for the 12S ARN gene.

PCR amplification was performed using a CFX96 Thermal Cycler (Bio-Rad Laboratories, Foster City, CA, USA) in a 20 μl reaction volume containing 5 μL of the DNA template, 10 μL of Eurogentec™ Probe PCR Master Mix (Eurogentec, Liège, Belgium), 0.5 μM of each primer and 0.5 μM of the FAM-labeled probe. The thermal cycling conditions included one incubation step at 50°C for two minutes and an initial denaturation step at 95°C for three minutes, followed by 40 cycles of denaturation at 95°C for 15 seconds and annealing extension at 60°C for 30 seconds.

At first, all the samples were screened in both qPCRs. Thereafter all those which were positive by at least one qPCR were subjected to conventional PCR targeting the 272 bps portion of *cytb* gene as described by Boutellis *et al*. [19].

All the products of the PCR amplification were checked on gel electrophoresis and then purified using NucleoFast 96 PCR plates (Macherey-Nagel EURL, Hoerdt, France) according to the manufacturer’s instructions. All products of both qPCRs and conventional PCR were sequenced with original primers on an ABI automated sequencer (Applied Biosystems, USA) with the BigDye Terminator v1.1 cycle (Applied Biosystems, Foster City, CA). The electropherograms obtained were assembled and corrected using ChromasPro software (Technelysium Pty, Queensland, Australia).

#### Detection of pathogens

To test for the presence of pathogens, qPCRs were performed using previously reported primers and probes targeting the 16S rRNA gene of *Borrelia* [29], the ITS intergenic spacer of *Bartonella* [8], the *rpoB* gene of *Acinetobacter* [9], the *ompB* gene of *Rickettsia prowazekii* and the *pla* gene of *Yersinia pestis* [27]. All qPCRs were performed using a CFX96 TMREAL-Time System C1000 Thermal Cycler (Bio-Rad Laboratories) and the Eurogentec Master Mix Probe PCR kit (Eurogentec).

#### Prevention of DNA contamination

In order to ensure that no contamination by modern DNA would interfere with the results, all pre-PCR and post-PCR procedures were performed respectively in a separate, clean room, free of louse DNA under a hood with air-capture, using autoclaved and UV treated material. Extractions and PCR amplification blanks were used as negative controls, in order to detect possible contamination by external DNA. No positive control was included in any experimental steps in order to minimize potential contamination. No amplifications were detected among the negative controls throughout the study.

### Data Analysis

MEGA 6.06 was used for phylogenetic analyses and tree reconstruction with 500 bootstrap replicates using the maximum likelihood method under Kimura’s 2-parameter with complete deletion [30]

For comparison, the ancient DNA sequences obtained in this study were combined with the 30 *cytb* haplotypes reported by Drali *et al*. [31]. This dataset was complemented with newly available sequences in GenBank, after being assigned to haplotypes using DnaSP v5.10 [32]. Ancient lice sequences from 10,000 and 4,000 year-old Peruvian and Chilean mummies, respectively, were also included [17, 26]. Finally, a dataset that consisted of 49 haplotypes was created in which two are shared between contemporary and ancient lice. These haplotypes span 40 geographic locations (countries) on five continents (S1 Table). The novel haplotypes identified were deposited in GenBank under the following accession numbers: KX249763-KX249775.

In order to investigate the possible relationships among haplotypes, the median-joining (MJ) network using the Bandelt method was constructed using the NETWORK4.6 program (www.fluxus-engineering.com/sharenet.htm) [33].

For *A*. *baumannii*, the nucleotide sequences obtained in this study were aligned with the reference sequences available in public databases (GenBank) and a phylogenetic tree was also constructed using the maximum likelihood method under Kimura’s 2-parameter model implemented in MEGA 6.0 6, with 500 bootstrap replicates [30].

## Results

In this study, we isolated DNA from twenty-four head lice parts and their eggs, *P*. *h*. *capitis*, from Roman-era remains retrieved in Israel. A 110-bp DNA fragment of the 12S RNA gene (qPCR) and 85-bp (qPCR) and 270-bp (conventional PCR) DNA fragments of *cytb* gene were targeted.

### Detection of Lice DNA in Ancient Lice Samples

The qPCR targeting an 85-bp fragment of *cytb* gene was positive for all the 24 ancient DNA lice tested (32<Ct<35), while only 22 samples of the 24 tested were positive for a 110-bp fragment of the 12S RNA gene (33<Ct<36) (S1 Fig).

### Determination of Lice Clade

To determine lice clade, all samples which were positive for at least one qPCR (24/24) were subjected to conventional PCR targeting a 272-bp fragment of the *cytb* gene coupled with sequencing (S2 Fig). To insure the reproducibility of the results, we also sequenced the products of both qPCRs, since the two shorts fragments amplified by both qPCRs can discriminate between all known clades of lice. Thus, at least one sequence per sample was successfully recovered from all the samples (24/24).

In comparison with previously well-defined lice mtDNA haplogroups A, C [16], B [1], D (*sensu* Drali et al.) [5] and E [20], all ten samples recovered from comb A belonged to mitochondrial clade B (41.6%). All lice samples from combs B (thirteen samples) and C (one sample) belonged to clade A (58.3%) (Table 1).

**Table 1:** Summary of ancient samples, DNA analyses and haplotypes assignment.

Louse number (Lab code)	Part of lice amplified	PCR results			Haplogroup identity	Haplotype identity on the basis of partial cytb 270-bp
		12S (110-bp)	Cytb (80-bp)	Cytb (270-bp)		
Comb A						
Romanic-HL1	thorax /abdomen	+	+	+	B	Hap_B36
Romanic-HL2	thorax /abdomen	+	+	+	B	Hap_B36
Romanic-HL3	Thorax	+	+	+	B	Hap_B36
Romanic-HL4	Abdomen	+	+	+	B	Hap_B36
Romanic-HL5	Abdomen	+	+	+	B	Hap_B36
Romanic-HL6	Abdomen	+	+	+	B	Hap_B36
Romanic-HN7	non operculated egg	+	+	+	B	Hap_B36
Romanic-HN8	non operculated egg	+	+	+	B	Hap_B36
Romanic-HN9	operculated egg	NA	+	NA	B*	—
Romanic-HN10	operculated egg	+	+	NA	B*	—
Comb B						
Romanic-HL11	entire male	+	+	+	A	Hap_A5
Romanic-HL12	entire nymph	+	+	+	A	Hap_A55
Romanic-HL13	leg/thorax/abdomen	+	+	+	A	Hap_A55
Romanic-HL14	thorax /abdomen	+	+	+	A	Hap_A5
Romanic-HL15	thorax /abdomen	+	+	+	A	Hap_A5
Romanic-HL16	thorax /abdomen	+	+	+	A	Hap_A5
Romanic-HL17	Thorax	+	+	+	A	Hap_A55
Romanic-HL18	Thorax	+	+	+	A	Hap_A5
Romanic-HL19	abdomen	+	+	+	A	Hap_A55
Romanic-HL20	abdomen	+	+	+	A	Hap_A5
Romanic-HN21	operculated egg	NA	+	NA	A*	—
Romanic-HN22	non-operculated egg	+	+	+	A	Hap_A5
Romanic-HN23	non-operculated egg	+	+	NA	A*	—
Comb C						
Romanic-HL24	entire nymph	+	+	+	A	Hap_A56
Total	24	22/24	24/24	20/24	14(A)+10(B)/24	7(A5)+4(A55)+ 1(A56)+8(B36)/24

NA: not amplified

*- clades identified on the base of cytb qPCR product sequencing

### Phylogenetic Analysis and Haplotype Assignment

**Fig 2:**
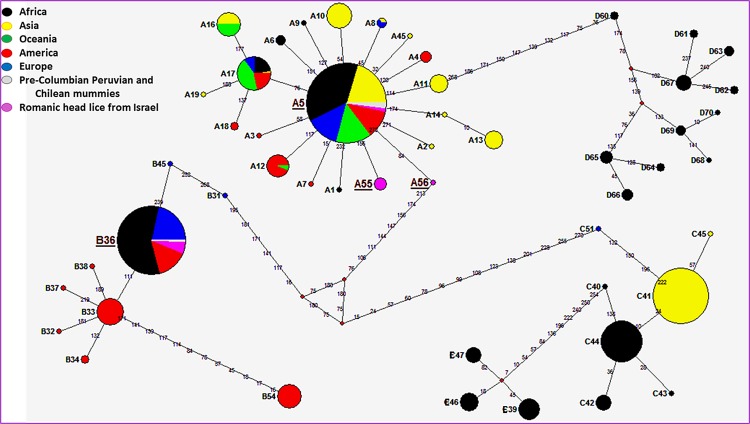
*Cytb* haplotype networks of contemporary and ancient human body and head lice. Each circle area indicates a unique haplotype and variations in circle size are proportional to haplotype frequencies. Pie colors and sizes in circles represent the continents and the number of their sequence for a haplotype. The length of the links between nodes is proportional to mutational differences.

**Fig 3:**
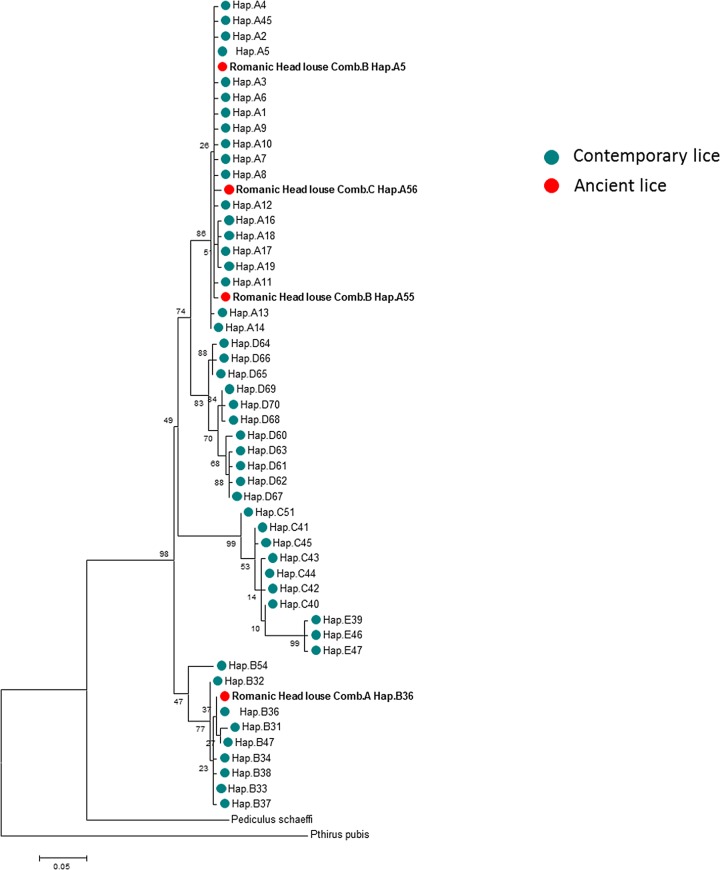
Maximum-likelihood (ML) phylogram of contemporary and ancient haplotypes of *Pediculus humanus* based on the partial 272-bp cytb gene with *Pediculus schaeffi* (KC241883) and *Pthirus pubis* (EU219990) as outgroups.

For the phylogenetic analysis we used the maximum likelihood method (ML) (Fig 2) and a median-joining (MJ) network (Fig 3) analysis. Only the 272-bp sequences of the *cytb* gene (20/24) were analyzed and combined with all known modern and ancient haplotypes generated in our dataset.

Within clade A (12/20), seven sequences (all from comb B) of these ancient head lice belonged to the widespread modern haplotype A5. This haplotype is the most prevalent worldwide (75% of locations and 45% of the 985 analyzed human lice) and is present in all continents. It occupies a central position in haplogroup A, as shown in the MJ network, and is also found in pre-Columbian Peruvian and Chilean mummies from the New World. The five remaining clade A sequences, four from comb B and one from comb C, yielded two novel haplotypes which were found to be unique, provisionally named here as A55 and A56, respectively. A thorough inspection of the haplotype network revealed that the A55 haplotype deviated from haplotype A5 by one mutation step while the A56 haplotype derived on haplotype A5 by two mutations steps. Nevertheless, the mutations that we found in both haplotypes A55 and A56 are synonymous, i.e. resulting in no amino acid changes, compared to the A5 haplotype. The sequences of these two novel haplotypes were deposited in GenBank under accession number: KX232678-KX232679.

Within the clade B ancient head lice, all eight sequences (all from comb A) belonged to the modern haplotype B36, which is the most common within the B-haplogroup (74% of the 184 analyzed haplogroup B human lice). This haplotype occupied a central position in the MJ network, and is found in Africa, Europe and America, as well as in pre-Columbian Chilean mummies.

### Detection of Pathogens

**Fig 4:**
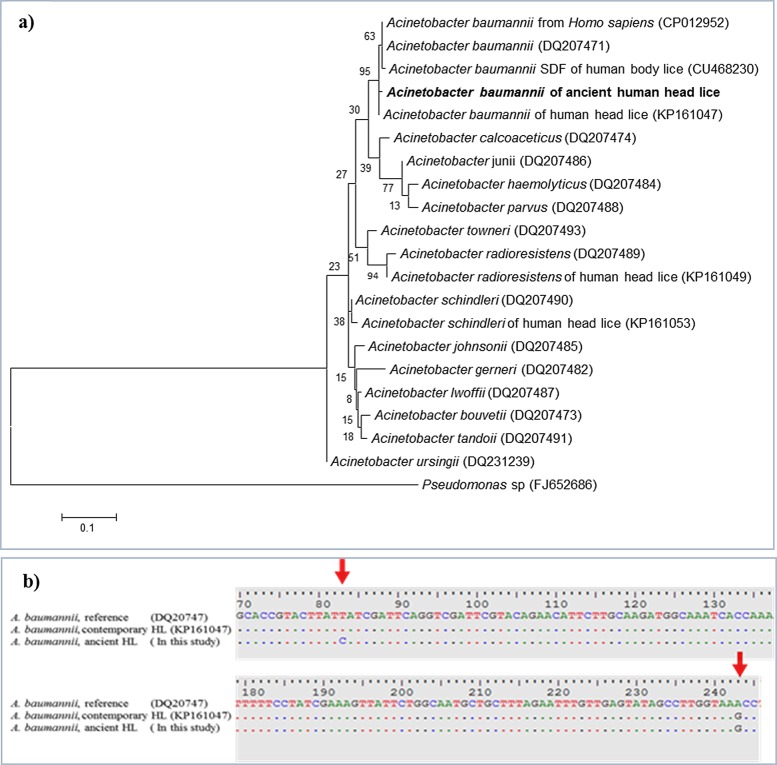
*Acinetobacter baumannii* from ancient head lice belonging to the Roman period. *a*, Maximum-likelihood (ML) phylogenetic tree relationship based on 182-bp fragment *rpoB* gene of *A*. *baumannii* detected in ancient head lice was compared with the reference sequences strain, while *Pseudomonas* was used as an out group. Bootstrap values are indicated at the nodes. Bold indicates the taxonomic position of *A*. *baumannii* identified in this study. *b*, 182-bp of *A*. *baumannii rpoB* gene fragment sequenced from the four ancient head lice, exhibiting one mutation not present in the homologous gene sequence from its closest relative, the modern *A*. *baumannii* sequence in GenBank”.

The qPCR targeting the *rpoB* gene of *Acinetobacter* was positive for four samples (4/24) of ancient lice DNA tested (34<Ct<36) from comb B. The qPCR products generated were directly sequenced. The four obtained sequences of the partial *rpoB* gene (182-bp) were 100% identical to one another and were identified as *A*. *baumannii* on the basis of a BLAST search. These sequences had 180 of 182 base positions in common (98.9% identity) with a reference strain of *A*. *baumannii* (GenBank accession number CP012952) and 181 of 182 base positions in common (99.4% similarity) with *A*. *baumannii* isolated from modern human head lice collected from elementary school children in Thailand (GenBank accession number KP161047). However, these ancient sequences have one mutation (position 81) that has not previously been described in modern *A*. *baumannii* (Fig 4B). The phylogenetic tree of this *A*. *baumannii* is shown in Fig 4A. All four *A*. *baumannii* positive ancient head lice belonged to clade A.

*Borrelia* spp., *Bartonella* spp., *Rickettsia prowazekii* and *Y*. *pestis* DNA could not be identified in any of the 24 ancient head lice specimens tested.

## Discussion

A paramount concern in studying ancient DNA is the prevention of its contamination by modern DNA [34]. Several elements support the authenticity of the results presented herein: qPCRs were designed specifically for this study, each experimental step was performed in separate rooms, free of lice and their DNA and no positive controls were used in the PCR examinations as recommended elsewhere [34]. Some of the sequences obtained were unique and presented mutations which have never previously been reported, lending greater confidence to the authenticity of the present results [34].

The mtDNA analysis in this study revealed that the head lice remains from approximately 2,000 years ago have *cytb* haplogroups A and B. To the best of our knowledge, the present study is the first to reveal the presence of a mitochondrial haplogroup B in a Middle Eastern region. It has thus far only been found in contemporary lice from America, Europe, Australia and, more recently, Africa (Algeria and South Africa) [2, 18, 19, 20]. Specifically, this haplogroup B has a B36 haplotype, the most common among B haplogroups [31]. Interestingly, this haplotype was also present for at least 4,000 years in South America before the arrival of European settlers [26].

Accordingly, the present findings show that clade B lice existed in the Mediterranean region long before the discovery of America, meaning that clade B lice did not originate from America as was previously thought but probably existed, at least in the Middle East, prior to contact between Native Americans and Europeans. These results are consistent with the hypothesis suggesting that this clade could originate from Asia, which is reported to have populated the Americas [1, 18].

The remaining samples had haplogroup A and yielded three haplotypes, in which two haplotypes (provisionally called A55 and A56 in this paper) were unique to the ancient head lice examined in this study, while haplotype A5, which is common worldwide, including in ancient head lice from Chilean and Peruvian mummies of the New World [17, 26, 31].

Prior research has suggested that the known lice clades evolved on different lineages of *Homo*, similarly to those known to date from 2.3 to 0.03 million years ago (MYA) [1, 20] and, accordingly, their geographic distribution can provide information regarding the evolutionary history of the lice as well as their human hosts [2]. Clade A lice most likely emerged in Africa and evolved on the host lineage that led to anatomically modern humans (*Homo sapiens*), showing signs of a recent demographic expansion out of Africa about 100,000 years ago, first to Eurasia and subsequently to Europe, Asia and the New World [1, 18]. Haplogroup B diverged from haplogroup A between 0.7 and 1.2 million years ago and may have evolved on archaic hominids, such as the *Homo neanderthalensis*, who expanded in Europe and Asia, and only became associated with modern humans during the period of overlap as the result of a recent host switch [1, 3, 4]. Thus, considering the present-day geographic distribution of the two haplotypes A5 and B36 and evidence of their long-time presence in the New World, along with our data from the Middle East, the most likely theory is that these two haplotypes were carried by early humans and migrated with them throughout the world before the globalization initiated during the time of Columbus. It seems that the B36 haplotype was originally present in archaic populations of the Middle East, and because this region was a passageway for *Homo sapiens* between Africa and the rest of the world [35], this haplotype could have switched to anatomically modern humans when they arrived, and migrated with them along with lice of haplotype A5 throughout the world including to America, where lice remained *in situ* for thousands of years until the second contact with European colonists in the 16^th^ century [2].

Aside from their role as biological markers used to infer human evolutionary history, lice remains can provide information relating to past human sanitary conditions and diseases, because these lice are vectors of bacterial pathogens. Yet, genetic traces from pathogens can be identified in archaeological remains and ancient DNA has successfully been used to identify *B*. *quintana* in 4,000-year-old teeth [36] and was reported to occur in lice at the end of World War I [37]. Raoult *et al*. [34] showed that Napoleon’s soldiers in Vilnius were exposed to body lice containing *B*. *quintana* and that soldiers had evidence of infection with either *R*. *prowazekii* or *B*. *quintana*, concluding that louse-borne infectious diseases affected nearly one-third of Napoleon’s soldiers buried in Vilnius, and indicating that these diseases might have been a major factor in the French retreat from Russia.

This study reveals for the first time the presence of *A*. *baumannii* DNA in ancient human head lice remains belonging to clade A. Specifically, the *rpoB* sequence that was found has not been reported previously.

In recent years, *A*. *baumannii* was first isolated from the body lice of homeless people in Marseille (France) as well as from diverse countries worldwide [38]. *A*. *baumannii* DNA was also found in head lice collected from elementary school children in Paris belonging to clade A [9] and detected in body and head lice collected from healthy individuals from Ethiopia [10]. In 2015, S. Sunantaraporn *et al*. showed that *A*. *baumannii* could be detected in clade A and C head lice collected from elementary school children in Thailand [13].

Although *A*. *baumannii* was reported to occur in temporary head lice in several occasions, the clinical significance of this finding is unknown [9, 39]. More recently, *A*. *baumannii* was shown to cause nosocomial infections and severe community-acquired infections such as pneumonia, bacteremia, endocarditis, and meningitis, due to its increasing resistance to a wide range of antibacterial agents [40, 41].

## Conclusion

The present work confirms that clade B lice existed, at least in the Middle East, prior to contacts between Native Americans and Europeans. Our results cannot support the previous hypothesis that clade B has an American origin and was imported from America to the Old World after its discovery by Christopher Columbus. We also identified the presence of nosocomial pathogen, *A*. *baumannii* in Roman-era head lice remains belonging to clade A.

Further study of the lice remains would be necessary to shed more light on the patterns of human migration worldwide, their lice and the flow of louse-borne pathogens at different times in history.

## Supporting Information

S1 Fig Real-time PCR amplification cuves for ancient DNA, *Pediculus humanus*, using *cytb* and 12S gens with negative controls (NC). 1, 2 and 3 showed qPCR amplification targeted a 88-bp DNA fragment of cytb gene (24/24 positive with Ct varied between 32 to 38); 4 showed qPCR amplification targeted a 100-bp DNA fragment of 12S gene (22/24 positive with Ct varied between 32 to 38).
(PPTX)

S2 Fig Agarose gel stained with syber safe showing the 277-bp amplification for *Pediculus humanus cytb* gene fragment obtained from ancient DNA with negative controls (T-). (PPTX)

S1 Table Geographic occurrences and frequencies of cytb haplotypes of human head and body lice. Haplotypes highlighted in blue are the newly identified haplotypes from sequences available in GenBank.
(XLSX)
