# Supplementary figures and images for: High Ancient Genetic Diversity of Human Lice, Pediculus humanus, from Israel Reveals New Insights into the Origin of Clade B Lice
Source: PLoS One. 2016 Oct 14;11(10):e0164659. doi: 10.1371/journal.pone.0164659 (PMC5065229; doi:10.1371/journal.pone.0164659)

## Slide 1
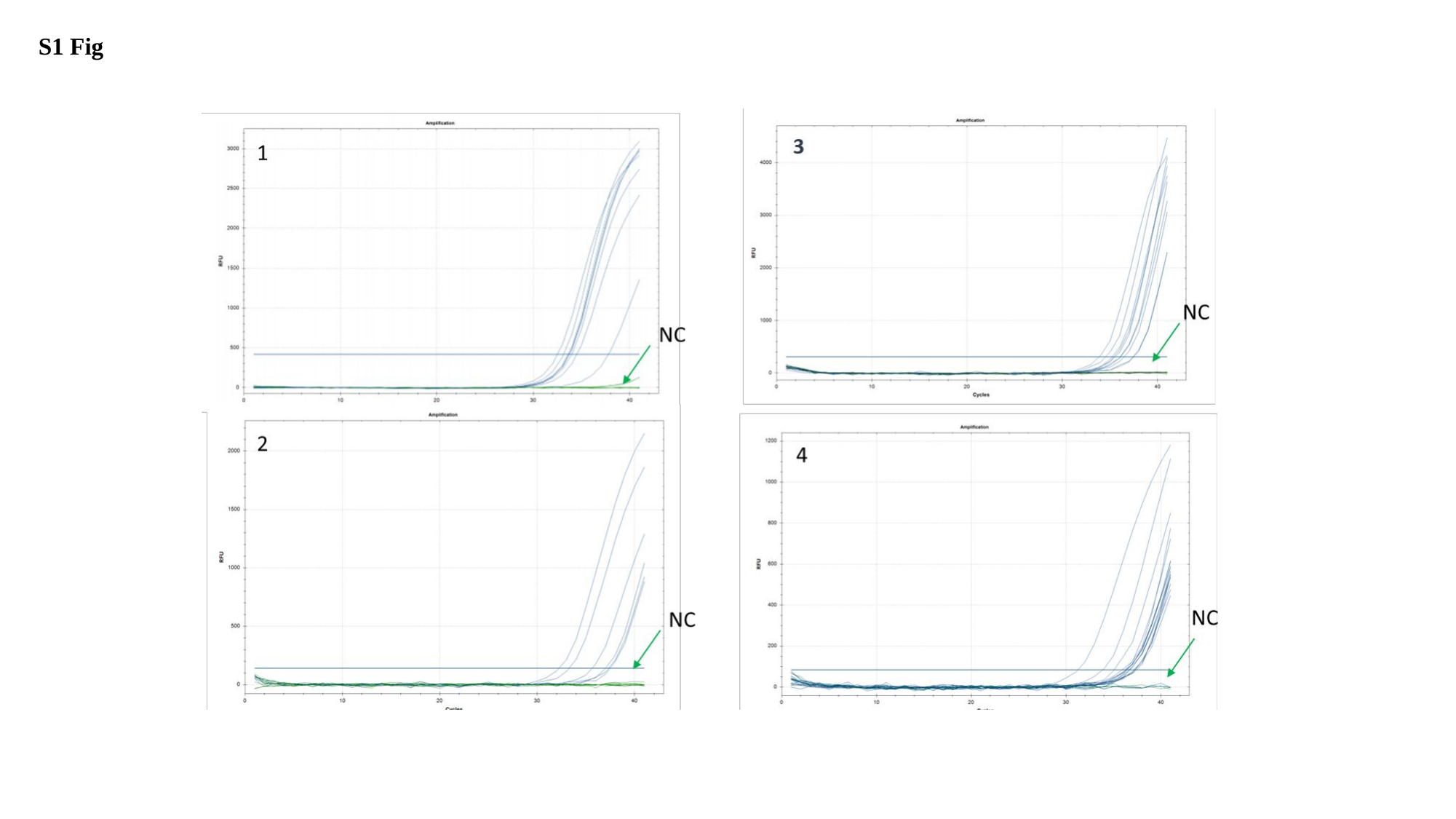

S1 Fig

Supplement: S1 Fig — 1, 2 and 3 showed qPCR amplification targeted a 88-bp DNA fragment of cytb gene (24/24 positive with Ct varied between 32 to 38); 4 showed qPCR amplification targeted a 100-bp DNA fragment of 12S gene (22/24 positive with Ct varied between 32 to 38). (PPTX) [file pone.0164659.s001.pptx]

## Slide 1
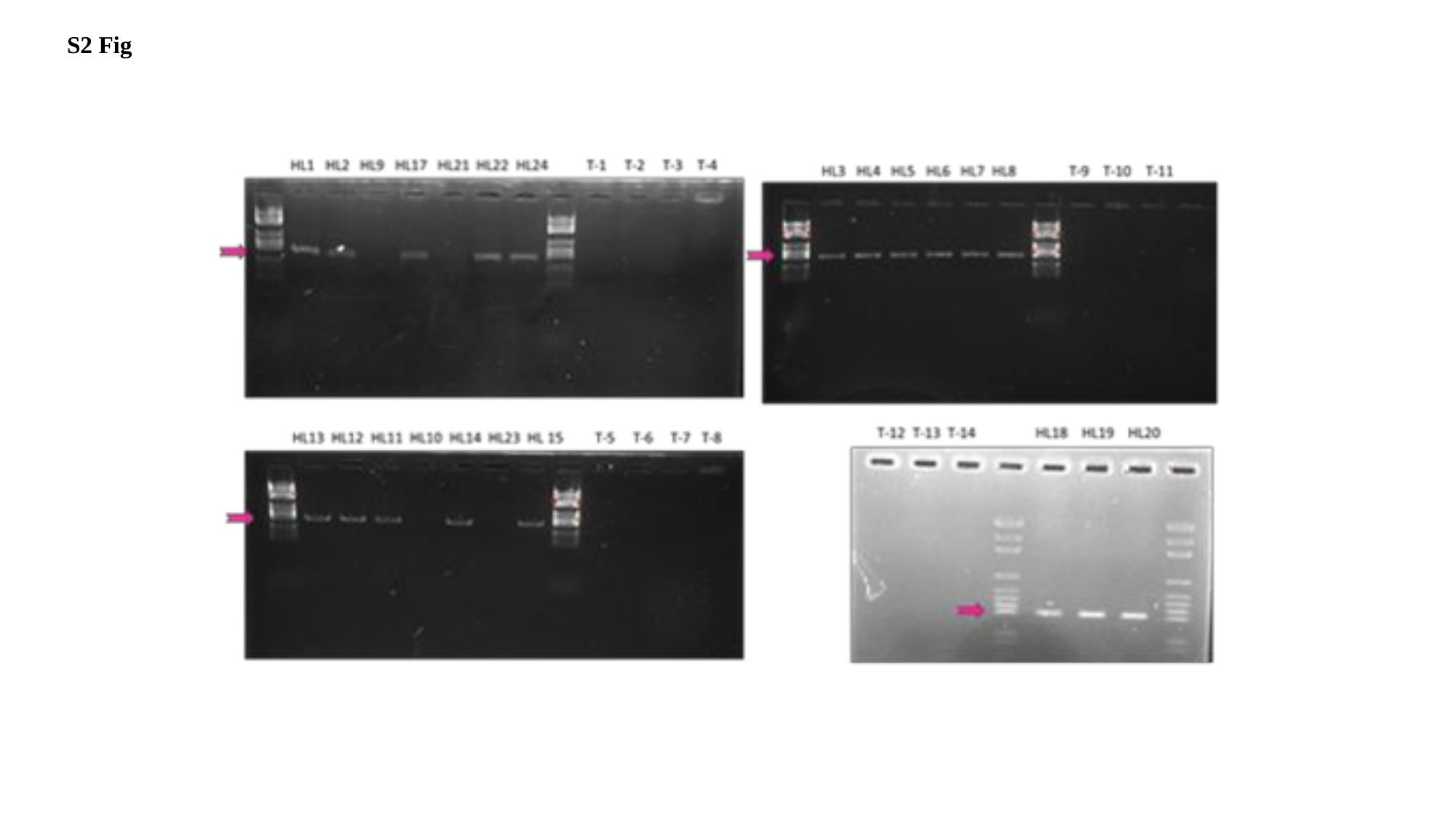

S2 Fig

Supplement: S2 Fig — (PPTX) [file pone.0164659.s002.pptx]
